# Advancements in and Research on Coplanar Capacitive Sensing Techniques for Non-Destructive Testing and Evaluation: A State-of-the-Art Review

**DOI:** 10.3390/s24154984

**Published:** 2024-08-01

**Authors:** Farima Abdollahi-Mamoudan, Clemente Ibarra-Castanedo, Xavier P. V. Maldague

**Affiliations:** Department of Electrical and Computer Engineering, Université Laval, 1065 Avenue de la Médecine, Quebec, QC G1V 0A6, Canada; xavier.maldague@gel.ulaval.ca

**Keywords:** coplanar capacitive technique, NDT methods, design parameters, defects identification, capacitive sensor

## Abstract

In contrast to conventional non-destructive testing (NDT) and non-destructive evaluation (NDE) methodologies, including radiography, ultrasound, and eddy current analysis, coplanar capacitive sensing technique emerges as a novel and promising avenue within the field. This paper endeavors to elucidate the efficacy of coplanar capacitive sensing, also referred to as capacitive imaging (CI), within the realm of NDT. Leveraging extant scholarly discourse, this review offers a comprehensive and methodical examination of the coplanar capacitive technique, encompassing its fundamental principles, factors influencing sensor efficacy, and diverse applications for defect identification across various NDT domains. Furthermore, this review deliberates on extant challenges and anticipates future trajectories for the technique. The manifold advantages inherent to coplanar capacitive sensing vis-à-vis traditional NDT methodologies not only afford its versatility in application but also underscore its potential for pioneering advancements in forthcoming applications.

## 1. Introduction

Ensuring the reliability of engineering components is vital to avoid early breakdowns. Non-destructive testing (NDT) methods, which can assist in assessing the integrity of materials without changing them, plays a key role in quality control during production and ongoing maintenance. These methods, which have been around since the early 20th century, find defects caused by production, the environment, or wear during service. They are cost-effective for inspecting individual components or entire parts during manufacturing. Advancements in microprocessors and automation have made defect monitoring even better, improving safety and efficiency while reducing the need for routine checks. NDT falls under non-destructive evaluation (NDE), which includes methods for spotting flaws on surfaces and inside materials without needing to take them apart [1,2].

The study of NDT methods reveals challenges in their practical application, such as requirements for couplants, heat or wave sources, and complex data analyzers, alongside limitations in coverage area, penetration depth, and result interpretation. A variety of NDT techniques have been developed, encompassing visual inspection (VI) or visual testing (VT) [3,4,5], ultrasonic testing (UT) [6,7,8,9], thermography testing (TT) or thermal imaging (TI) [10,11,12,13,14], radiography testing (RT) [15,16,17], eddy current testing (ECT) [18,19,20,21], acoustic emission (AE) [22,23,24], and shearography testing [25,26,27], each with distinct advantages and limitations. These methods aim to identify potential hazards, damage, or defects in environments, manufactured goods, and structures, ensuring safety compliance. Comprehensive assessments of their capabilities, limitations, advantages, and disadvantages can be found in the pertinent literature [28,29,30,31,32,33].

In contrast to the well-developed NDT methods available for metallic materials, those for non-conducting materials are comparatively less advanced. One method for evaluating non-conducting materials involves characterizing their dielectric properties, specifically focusing on parameters such as dielectric strength and dielectric constant. Dielectric strength refers to the voltage threshold a material can endure before experiencing electrical breakdown, while dielectric constant, also known as permittivity, measures the material’s ability to store electric energy. Alternative approaches, such as microwave techniques and resonant testing, exist for characterizing materials’ permittivity, albeit often requiring costly equipment and/or intricate operational procedures. In contrast, capacitive sensing presents a straightforward and cost-effective approach to assessing structures, especially non-conducting materials [1]. The capacitive sensing technique offers promise for evaluating materials like composites, insulated pipes, concrete structures, and wire insulation, providing an alternative approach to address the limitations of traditional techniques.

The capacitive sensing technique, established as the NDT method in 2006, as the NDT method, represents a significant advancement in non-destructive inspection methodologies. This non-contact and non-invasive approach, as pioneered by Liu and Liu [34], has emerged as a promising avenue within the realm of NDT. Referred to as capacitive imaging (CI), it stands as an electromagnetic NDT method [35], distinguished by its utilization of arrays of electrodes to engender an alternating current (AC) electric field distribution within the material under examination. This distribution can permeate dielectric materials, responding dynamically to the material’s internal structure. By scanning these electrodes across the specimen, variations in the electric field distribution yield alterations in the output voltage. Notably, the capacitive technique boasts versatility in inspecting a diverse array of materials and structures, ranging from insulators to conductors, without the drawbacks commonly associated with traditional methods, which are restricted to particular materials exhibiting distinct physical characteristics [36]. The efficacy of this methodology hinges upon the intricate interplay between electric fields and structural variations within the material, a principle elucidated by the seminal works of Bozzi and Bramanti [37] and Yin et al. [35]. Remarkably, the coplanar electrodes employed in capacitive coupling facilitate deep penetration into non-conducting materials while retaining applicability to those possessing reasonable conductivity levels. Such attributes position the capacitive technique as a promising paradigm within the NDT domain, offering enhanced capabilities for material inspection and structural integrity assessment [38].

This paper delves into the examination, assessment, and advancement of coplanar capacitive sensing methodologies for non-destructive material inspection. To lay the groundwork for this review, an extensive literature review was undertaken, encompassing a range of scholarly articles. Through this comprehensive inquiry, various benefits, current obstacles, and areas requiring further exploration were unearthed, thus providing valuable insights into potential avenues for future development. Through an extensive survey of the NDT review literature, it became evident that the capacitive testing technique is notably underrepresented in comparison to other NDT methodologies discussed in these articles. Despite its compatibility with applications where conventional techniques face limitations, it lacks adequate coverage in the existing literature. Consequently, the main purpose of this paper is to provide a comprehensive review elucidating the utilization of coplanar capacitive sensors for defect detection within the realm of NDT, aiming to underscore their efficacy through an extensive synthesis of the existing literature in the field.

The structure of this review paper is meticulously outlined to ensure a comprehensive exploration of coplanar capacitive sensing techniques in NDT. The exposition begins with the fundamental concept of the coplanar sensor, detailing its design principles, fabrication processes, and the underlying physics governing its operation. This foundational segment sets the stage for subsequent technical discussions. Following this, the discussion delves into numerical simulation and foundational theory calculation, employing advanced computational techniques such as the Finite Element (FE) method to analyze and predict the sensor’s behavior under various conditions. The discussion then progresses to cover the penetration depth and influential factors affecting sensor performance. Key parameters such as the geometry, dimensions, and spacing of electrodes, as well as the impact of shielding plates, guard electrodes, lift-off distance, and operational frequency on the sensor’s efficacy, are examined. This section provides a detailed analysis of how these factors influence the sensor’s ability to detect defects at different depths and in varying material conditions. Subsequently, the application of coplanar capacitive sensing to defect detection applications is thoroughly examined. The practical advantages and limitations of this technique are explored, highlighting its sensitivity, spatial resolution, and adaptability to different types of defects and materials. This part of the review is enriched with case studies and examples demonstrating the sensor’s performance in real-world scenarios. Finally, the conclusive section synthesizes the extant challenges, issues, and future prospects pertaining to the coplanar capacitive sensing technique in NDT. Current technological barriers, potential areas for improvement, and emerging trends that could shape the future development and application of coplanar capacitive sensors are discussed. This forward-looking analysis aims to provide valuable insights for researchers striving to advance the field of NDT through innovative sensing solutions.

## 2. Fundamental Concept of the Planar Arrangement

**Figure 1:**
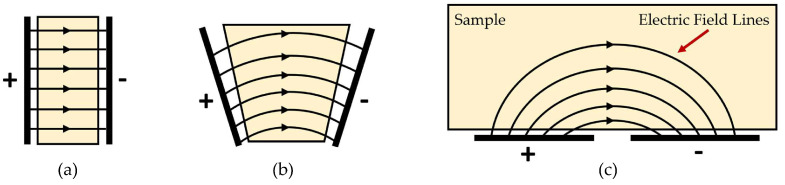
Diagram illustrating the transition of electric field distribution as electrodes shift from parallel geometry to coplanar: (**a**) Parallel-plate capacitor, (**b**) Electrodes diverge, and (**c**) Single-sided access to the sample [42].

The fundamental premise of this technique entails the strategic placement of two or more electrodes onto the surface of a test specimen, followed by the application of an alternating current (AC) voltage across them. This configuration effectively forms a capacitor, wherein variations in capacitance serve as indicators of internal structural characteristics, including the detection of defects [38]. In conventional capacitor configurations, plates are typically aligned in parallel (see Figure 1a), facilitating the generation of a uniform electric field distribution upon voltage application. However, as the electrodes gradually separate, the electric field expands beyond the confined space between them, resulting in the formation of a fringe field that extends into a broader region [39,40]. This phenomenon, known as fringing, is pivotal for imaging purposes as it enables the penetration of the fringe field into the sample. By systematically scanning a pair of electrodes across the specimen’s surface and meticulously measuring changes in stored charges at a given voltage, a comprehensive map of alterations in the electrical properties within the specimen is meticulously delineated [41]. Upon transitioning the electrodes to a coplanar orientation (see Figure 1c), the prominence of the fringe field between the driving and sensing electrodes becomes apparent. The driving electrodes in coplanar capacitive sensing are typically responsible for generating an electric field that interacts with the material under test. These electrodes are designed to induce changes in capacitance based on the material’s dielectric properties. On the other hand, sensing electrodes are configured to detect these changes in capacitance, thereby converting them into measurable signals. The specific criteria would be used to select the dimensions, materials, and configurations of the driving and sensing electrodes to optimize performance in non-destructive testing applications. The configuration in Figure 1c, commonly referred to as a coplanar sensor in the academic literature [40], is depicted schematically in Figure 1 [42], illustrating the evolution of electric field distribution from a parallel plate to coplanar configuration, resulting in the production of a fringing field [41].

It is worth mentioning that the impact of media homogeneity on the sensor accuracy should be considered. In homogeneous media, the consistent dielectric properties ensure stable electric field distribution, resulting in accurate and reliable measurements. The coplanar capacitive sensing technique excels in such environments due to its sensitivity to small capacitance changes. In contrast, non-uniform media can disrupt the electric field propagation, leading to errors. Variations in the dielectric constant cause the electric field to refract, reflect, or scatter, affecting the sensor’s ability to measure capacitance changes accurately [43]. Enhancing the sensor design, such as incorporating shielding and guard electrodes, can help improve measurement stability in non-uniform media. These modifications can reduce the influence of external factors and focus the sensing region more precisely [42]. Consequently, while the coplanar capacitive sensing technique is more suitable for homogeneous media due to the stable propagation of electric fields, it can still be effectively applied to non-uniform media with appropriate considerations and adjustments. Acknowledging these limitations and incorporating strategies to address them will enhance the robustness and versatility of this sensing technique in a wider range of applications [44]. In addition, signal distortion is a critical issue in sensor technology, affecting the accuracy and reliability of measurements. The Bayesian multiple linear regression (BMLR) method addresses this problem effectively. Signal distortion can arise from various sources, such as data time lag and abnormal signals like drift and jump points, which are common in the long-term monitoring of structures. To counteract signal distortion, the article introduces a new modeling paradigm using BMLR. This method is robust to data time lag and abnormal signals by dynamically updating the model based on Bayes’ theorem, allowing for continuous improvement as new data are collected. Unlike traditional methods, BMLR can handle sparse sensing points and short-term observation data, making it suitable for environments where sensor data may be incomplete or delayed. For further reading and a deeper understanding of methods to mitigate signal distortion in sensors, you can refer to the literature on Bayesian approaches and their application in structural health monitoring, such as the article available in [45].

The inherent advantage of the coplanar structure lies in its capability to facilitate specimen inspection from a singular side, a feature particularly beneficial in scenarios where access to both sides of the specimen is constrained. This characteristic is notably advantageous in examining large mechanical structures or components situated within intricate assemblies, where conventional inspection methodologies may prove impractical or infeasible [46]. Moreover, the coplanar arrangement enhances the precision and efficiency of surface or intrinsic property inspection. By traversing the sensor electrodes over the surface of the structure, comprehensive data regarding its integrity, composition, and performance characteristics can be meticulously gathered [47]. This capability not only streamlines the inspection process but also enables proactive maintenance and quality control measures, thereby bolstering the reliability and longevity of critical engineering assets.

**Figure 2:**
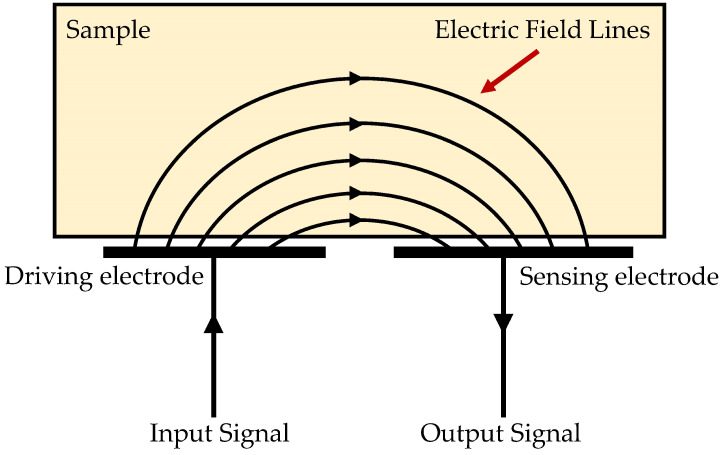
Diagram depicting the operational concept of the coplanar capacitive sensor: sensor examining a solid non-conductive sample [50].

The conceptual framework of the technique is inherently straightforward, elucidated by a fundamental diagram exemplifying its operational principles, as depicted in Figure 2, wherein coplanar electrodes maintain a consistent distance from and parallel orientation to the surface of the specimen under scrutiny [36]. Upon application of an alternating current (AC) voltage across the electrodes, an electric field distribution is established within the specimen. Any alteration in the specimen’s properties, such as the presence of a defect, within the region encompassed by the electric field distribution induces changes in the field pattern [48]. These variations in the electric field, in turn, prompt modifications in the charge stored between the sensor electrodes, consequently affecting the capacitance between them and, thereby, modulating the output voltage. This resultant output voltage serves as a discernible indicator of the specimen’s properties, encompassing parameters such as permittivity, conductivity, and their respective spatial distributions, as well as system variables like moisture and temperature, which bear a direct correlation with these properties [36]. Notably, deviations in the electric field, precipitated by factors such as delamination, cracks, or other imperfections, engender alterations in the induced charge at the sensing electrode, thus furnishing a discernable signal that can be harnessed for defect detection purposes [49]. This signal, subsequently relayed to standard instrumentation such as charge amplifiers, facilitates the precise recording, processing, and translation of minute variations in the output signal into a direct current (DC) voltage output. Given the diverse ways in which defects perturb the electric field distribution, a foundational framework for defect detection is established, providing a robust basis for discerning imperfections within the specimen [47].

The fabrication process for the sensor encompasses meticulous steps, commencing with the careful selection of materials for the electrodes, the insulation layer atop the electrodes’ surface, and the sensor substrate, and culminating in the choice of a suitable production methodology. Electrodes, typically composed of highly conductive materials such as copper, undergo stringent material selection to ensure optimal performance. A thin insulation layer, typically measuring a few micrometers in thickness, is thoroughly applied to the electrode surface to prevent direct contact with the specimen and shield against surface abrasions. The thickness of both the insulation layer and sensor substrate critically influences the electric field strength and depth of penetration, necessitating meticulous optimization. Various manufacturing techniques, including microelectromechanical systems (MEMSs) [51], printed circuit boards (PCBs), and manual production methods [52], are considered based on sensor dimensions and cost constraints [40]. Furthermore, cables and connectors play a critical role in the integrity of a measuring system, often representing its most vulnerable components. Movements, such as bending or tension, in the coaxial cables interconnecting the coplanar capacitive probe, charge amplifier, and lock-in amplifier can induce charge transport and local changes in capacitance. Consequently, the resulting charge signal stemming from cable movement becomes indistinguishable from the probe output, posing challenges, particularly when measuring small features. To mitigate this issue, it is imperative to minimize cable length and prevent relative motion between cables, aside from the necessary slack required for the probe’s free movement [53].

## 3. Numerical Simulation and Foundational Theory Calculation

Scientists and engineers create conceptual and mathematical models to understand various phenomena and systems. This understanding aids in the development and enhancement of systems that increase human convenience and comfort. Mathematical models are constructed based on axioms or natural laws governing the phenomena. These models typically comprise algebraic, differential, and/or integral equations. However, solving these equations for desired system variables can be challenging due to various input parameters, also known as data. Consequently, numerical simulations are often employed to analyze the phenomena [54,55]. To explore the coplanar capacitive sensing technique, the FE method is predominantly selected for analyzing the electric field between the sensor’s electrodes. Moreover, it is acknowledged that Conformal Mapping approaches, specifically the Schwarz–Christoffel mapping [56], can also serve as viable methods for this analysis.

The FE method is a powerful computational tool widely used in engineering disciplines, including civil, mechanical, and, increasingly, electronic engineering, for analyzing complex structures and systems. The FE method involves discretizing a large system into smaller, simpler parts called finite elements. This method provides numerical solutions to complex problems involving stress, heat transfer, fluid dynamics, and more. The application of the FE method in electronic engineering may not be as historically extensive as in civil or mechanical engineering, but its use has been growing due to the complexity of modern electronic devices and systems. For instance, the FE method is utilized in the design and optimization of electronic components to predict thermal behavior, electromagnetic fields, and mechanical stresses, which are critical for ensuring reliability and performance. The methodology’s flexibility and accuracy in modeling complex physical phenomena make it an invaluable tool in the design and analysis of modern electronic systems [57]. In this method, the domain under consideration is divided into subdomains, with each subdomain’s governing equation being approximated using traditional variational methods. The rationale behind seeking an approximate solution across subdomains lies in the ease of representing complex functions as collections of simple polynomials. The domain’s geometry can be discretized based on its shape, resulting in a mesh composed of various types of elements, such as rectangles and triangles, especially in irregular domains. However, the interfaces between elements must maintain compatibility to ensure a continuous solution [54,58,59].

**Figure 3:**
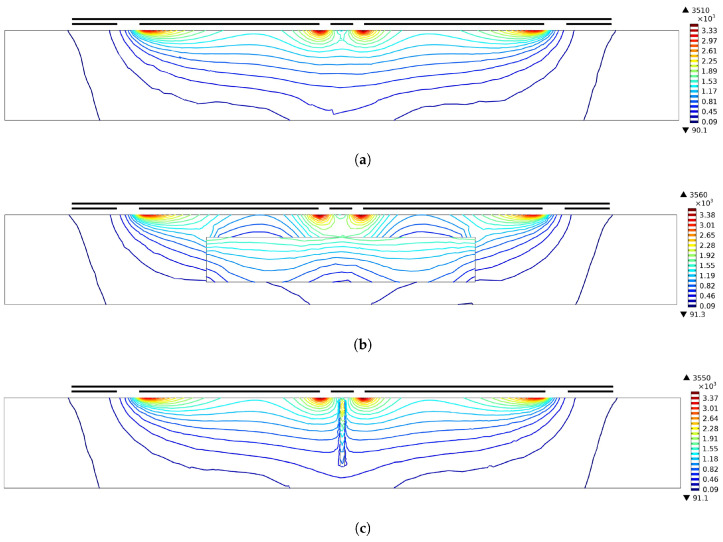
Simulations were conducted to analyze the distribution of the electric field (V/m) for different conditions: (**a**) without defects, (**b**) with a void beneath the surface, and (**c**) with a narrow crack on the surface of the sample [52].

Furthermore, FE modeling proves to be a valuable tool for predicting fields emanating from coplanar capacitive electrodes and assessing their interactions with diverse specimens, including those with various flaws. Extensive research has delved into the application of the FE method for field predictions and estimating signal variations [35]. Theoretical simulation models were developed using the COMSOL™ Multiphysics Finite Element package, specifically utilizing its AC/DC module, Electric Currents (EC), which operates in the three-dimensional (3D) frequency domain. The AC/DC Module within COMSOL Multiphysics offers a distinct environment tailored to simulating AC/DC electromagnetics in both 2D and 3D domains. This module serves as a robust tool for a detailed analysis of electrical machinery, facilitating static simulations through an intuitive graphical user interface. Figure 3 illustrates the predicted electric field distribution using 3D FE models for coplanar capacitive electrodes under various conditions. The figure presents contour plots of the electric field: Figure 3a depicts a sound zone of the specimen, Figure 3b shows an air-filled defective zone, and Figure 3c illustrates the electric field distribution for a specimen with a narrow surface crack. These simulations demonstrate that defects alter the electric field both within and around regions of discontinuity in the sample, resulting in variations in the detectable signal at the sensing electrode. This phenomenon arises from differences in dielectric properties, specifically permittivity (or dielectric constant), between the specimen and defects. Thus, the model elucidates how capacitive sensors detect these variations to identify defects in materials. The subsequent section provides an overview of the theoretical underpinnings of the AC/DC Module, which is Maxwell’s Equations, aiming to elucidate the underlying processes when employing the physics interfaces.

At its core, the complex interplay of electromagnetic phenomena finds its foundation in Maxwell’s equations, which serve as the bedrock of understanding in this domain. In instances where materials demonstrate a combination of dielectric and conductive characteristics, the Maxwell–Ampere equation emerges as a pivotal framework for analysis and comprehension [41]:

(1) $$\nabla \times H=J+\frac{\partial \epsilon_{0}E}{\partial t}=J+\frac{\partial D}{\partial t}$$

This equation encapsulates the dynamics governing the interaction between electric currents and the magnetic fields they induce, offering a comprehensive perspective on the electromagnetic behavior exhibited by materials possessing diverse electrical properties. In the equation presented, where ***H*** represents the magnetic field intensity, ***J*** denotes the free current density, $\epsilon_{0}$ signifies the electric constant or the permittivity of vacuum, ***E*** signifies the electric field intensity, and ***D*** represents the electric flux density. To mitigate the influence of the magnetic field intensity ***H***, the divergence operation is applied to both sides of Equation (1), yielding Equation (2):

(2) $$\nabla \cdot (J+\frac{\partial D}{\partial t})=0$$

Prior research has postulated that within the frequency range typically employed for coplanar capacitive sensors, namely, in the kilohertz (kHz) range, the distribution of electric fields within dielectric materials characterized by low electrical conductivity (such as ceramics, polymers, insulation materials, etc.) closely resembles that observed in the direct current (DC) or electrostatic scenario. Consequently, inductive phenomena are deemed negligible, permitting the electromagnetic field to be regarded as a quasi-static electric field, wherein current induction within the sample can be disregarded. This implies that the time-derivative of the magnetic flux density (***B***) is considered insignificant, thereby rendering the electric field (***E***) as an irrotational field, as postulated by Faraday’s Law [35,41]:

(3) $$\nabla \times E=-\frac{\partial B}{\partial t}=0.$$

In accordance with Equation (3), the electric field (***E***) may be delineated by an electric scalar potential distribution denoted by *V*(x, y, z). Thus, the electric potential distribution *V*(x, y, z) is introduced as elucidated by Yin and Hutchins [41]:

(4) E=−∇V(x,y,z)

And utilizing the constitutive relationships:

(5) J=σ(x,y,z)E,

(6) D=ϵ(x,y,z)E.

Equation (2) may assume the following expression:

(7) $$\nabla \cdot \left[\sigma \left(x,y,z\right)\nabla V\left(x,y,z\right)\right]+\nabla \cdot \left[\frac{\partial \epsilon (x,y,z)\nabla V(x,y,z)}{\partial t}\right]=0.$$

In the context provided, σ (x,y,z) denotes the conductivity distribution and ϵ (x,y,z) signifies the permittivity distribution. Should the distributions of conductivity and permittivity within the region under consideration, enveloped by the electric field, be ascertainable, the electric potential distribution *V* (x,y,z) can be derived by solving Equation (7). However, in practical applications, the computational complexity arising from the temporal coupling between dielectric and conductive properties renders the direct solution of Equation (7) prohibitive. Consequently, a pragmatic approach involves treating the system as either “predominantly dielectric” or “predominantly conductive” [60]. In the former scenario, Equation (7) lends itself to simplification, as discussed by Yin and Hutchins [41]. Specifically, the mathematical model governing the electrical potential *V* (x,y,z) conforms to Laplace’s equation, as expounded by Yin et al. [35]:

(8) ∇·[ϵ(x,y,z)∇V(x,y,z)]=0.

Upon the application of electric potentials to a sensing system comprising sensor electrodes and a specimen, a distinct set of boundary conditions is delineated, as explicated by Hu and Yang [40]. These pertinent boundary conditions, as outlined by Yin et al. [35], encompass

$$V(x,y,z)=\left\{\begin{array}{c} \phi |\left(x,y,z\right)\in \Gamma_{1}\;\;\;\;\;\;\;\;\;\\ 0|\left(x,y,z\right)\in \Gamma_{2}\;\;\;\;\;\;\;\;\;\;\;\;\;\; \end{array}\right.$$

The region $\Gamma_{1}$ delineates the domain encompassing the external surfaces of the driving electrode, where a voltage ϕ is established. Meanwhile, $\Gamma_{2}$ denotes the outer boundary delimiting the area covered by the FE model. Subsequently, the FE model facilitates the prediction of field variations within this defined domain. Although the electric potential distribution can theoretically be derived from Equation (8), this equation represents a complex potential function whose solution eludes straightforward attainment. Consequently, the FE method emerges as a practical recourse for predicting solutions to this equation. In practice, Equation (8) is discretely solved within finite domains, with each element representing discrete units. Leveraging the quasi-static assumption aforementioned, the FE technique serves as a viable approach to solving the aforementioned equations and forecasting the potential distribution *V* (x, y, z) generated by capacitive electrodes within specific media and geometries. Subsequent to obtaining the potential distribution *V* (x, y, z), Gauss’s Law, formulated in a numerical integral form, facilitates the computation of induced charge on the sensing electrode. Additionally, Gauss’s law, expressed in numerical integral form, can be employed on the surface enclosing the sensing electrode to compute the induced charge resulting from the electric potential distribution, should such analysis be required. This application of Gauss’s law is extensively discussed in [35,41]:

(9) q=−ϵ(x,y,z)∇V(x,y,z)ds

where *s* represents the surface encompassing the sensing electrode. It can be deduced through an analysis of Equations (7)–(9) that, for a given driving signal, the magnitude of charge on the sensing electrode is exclusively dictated by the electrical properties exhibited by the materials within the field, as dictated by the permittivity and conductivity distributions σ (x, y, z) and ϵ (x, y, z), respectively. Consequently, alterations in the structure or composition of materials within a specimen, which influence the permittivity and conductivity distributions, manifest as variations in the total charge on the sensing electrode, thereby reflecting as changes in the ultimate outcome. Remarkably, the capacitive technique demonstrates the capability to discern both surface cracks and sub-surface discontinuities, as elucidated in [41].

## 4. Coplanar Sensor Penetration Depth

In the realm of coplanar capacitance probes, the concept of penetration depth assumes paramount significance, denoting the maximum distance in the z-direction wherein the sensor elicits a discernible alteration in its output signal, thus signifying its sensing capacity and delineating the depth at which changes in electrical properties can be reliably detected [61,62]. This pivotal parameter is contingent upon several factors, notably the permittivity of the material under examination, the noise characteristics inherent in the instrumentation system, and crucially, the design attributes of the probe itself, encompassing its geometric configuration, dimensions, and electrode separation distance [43,63]. While it is acknowledged that the decay rate of the electric field from a given driving electrode is accelerated in materials characterized by a higher permittivity, it is the intricacies of the probe design that predominantly dictate the ultimate penetration depth achievable for a given material [43,46].

Notably, delineating the precise depth of penetration for coplanar capacitive sensors proves to be a complex endeavor owing to the intricate nature of the fringing electric field and the diverse array of probe geometries encountered. In recognition of this inherent complexity, a consensus remains elusive regarding standardized methodologies for determining penetration depth. Nevertheless, one approach involves assessing the effective penetration depth by systematically positioning a sample beneath the probe, subsequently incrementally displacing it away from the surface while monitoring the resultant output signals across the driving and sensing electrodes [40]. This methodological framework provides a practical means of evaluating the depth at which the sensor is capable of detecting changes in electrical properties, thereby offering valuable insights into its operational capabilities and informing the optimization of probe design parameters for enhanced sensing performance.

## 5. Influential Factors of Sensor Performance

In coplanar capacitive sensor design, various parameters play crucial roles in assessing sensor performance. Each design parameter possesses the potential to influence multiple aspects of sensor functionality, while simultaneously considering multiple design factors becomes imperative to attain the desired sensor performance. Consequently, understanding the impact of design parameters on sensor functionality facilitates the optimization of sensors tailored to specific applications. Furthermore, addressing instrumentation-related concerns is essential to ensure accurate sensor measurements [40,64]. Among the key design parameters for a coplanar capacitive sensor are electrode geometry, electrode count, their spatial arrangement, as well as the inclusion of shielding plates and guard electrodes [40,65]. These parameters influence detection sensitivity, accuracy, signal strength, and penetration depth, which are the primary criteria for evaluating the performance of a coplanar capacitive sensor [66].

Detection sensitivity is a crucial performance metric in coplanar capacitive sensing, determining the sensor’s ability to detect small changes in the dielectric environment or surface conditions of a material under test. In this context, sensitivity refers to the capability of the sensor to discern minute variations in capacitance, which are induced by factors such as material defects, surface irregularities, or changes in the dielectric constant of the sample. High sensitivity in coplanar capacitive sensors allows for the detection of subtle anomalies that may indicate structural flaws or variations in material properties. This sensitivity is particularly advantageous in NDT applications where precise identification of defects, cracks, or delaminations in materials is essential for ensuring structural integrity and reliability [40,67].

Accuracy is a fundamental characteristic of coplanar capacitive sensors, representing the degree of agreement between the sensor’s measured values and the true or accepted values of the quantity being measured. In the context of coplanar capacitive sensing, accuracy refers to how closely the sensor can determine the actual capacitance changes induced by variations in the material under test, such as defects, thickness variations, or changes in dielectric properties. High accuracy in coplanar capacitive sensors is crucial for a reliable detection and quantification of small changes in capacitance, ensuring a precise assessment of material conditions in NDT applications, quality control, and structural health monitoring. This capability allows for the detection of subtle defects and abnormalities that could compromise the performance or safety of structures or components [42,68].

Signal strength in the context of coplanar capacitive sensors used for NDT applications refers to the magnitude of the electrical signal generated by the sensor in response to the presence of a target material or defect. This parameter is critical for the sensor’s ability to detect, characterize, and quantify material properties or defects. Essentially, signal strength determines how effectively the sensor can detect changes in the material being tested, such as variations in permittivity, thickness, or the presence of voids and cracks. A higher signal strength typically implies better detection capability, leading to more accurate and reliable measurements [43,63].

Accordingly, the performance of coplanar capacitive probes is primarily dictated by the geometric configuration of their electrodes. These configurations range from simple symmetrical designs, such as pairs of triangular, square, or rectangular shapes, to more intricate forms like comb-shaped (interdigital) arrangements. Another prevalent geometry is the concentric configuration, which may feature a central disc surrounded by multiple outer annuluses or a series of concentric rings [40,53,69,70]. Crucial parameters for assessing capacitive sensor performance include electrode size, inter-electrode separation distance, the presence of shielding plates, and the incorporation of guard electrodes, all of which significantly influence penetration depth and electric field strength. These factors are paramount considerations in evaluating capacitive sensor efficacy, and elucidating the impact of each parameter on sensor performance is imperative for optimizing sensor design and achieving desired outcomes [43].

Numerous studies have addressed the primary parameters governing coplanar capacitive sensor performance across various configurations. For example, in [44], the FE method was employed to explore the effects of electrode width and separation distance for a pair of rectangular electrodes within a two-dimensional (2D) model that did not consider electrode length variations, nor did it incorporate shielding plates or guard electrodes. Similarly, [35] assessed the impact of electrode size using equilateral triangular electrodes but did not individually consider the effects of separation distance or shielding plates. In Reference [43], the study focused on the separation between the centroids of square electrodes, the presence of guard electrodes for rectangular electrodes, and the influence of shielding plates for triangular electrodes, yet it did not address electrode size. Reference [71] examined the effects of different triangular electrode sizes across three probes while maintaining constant electrode separation, thereby neglecting an analysis of this parameter. Furthermore, [72] investigated three pairs of triangular probes with varying areas but did not individually explore the effects of electrode size and separation distance. Comprehensive investigations have been conducted for concentric geometry [40,43] and symmetrical geometry [42] to examine the effects of each parameter, including electrode size, separation distance, shielding plates, and guard electrodes. The subsequent sections entail an examination of the individual impact of each parameter on the performance of coplanar capacitive sensors.

### 5.1. Geometry of the Electrodes

Different geometric configurations generate electric fields characterized by distinct sensitivity distributions, signal strengths, and penetration depths. Each shape influences how the electric field propagates and interacts with its surroundings, impacting its effectiveness in various applications [73]. Various shapes of electrodes, including rectangular, square, circle, comb-shaped, and concentric rings, have been studied, as reported in several papers [34,35,39,40,43,63,70,71,74,75,76,77]. In a seminal study referenced as [42], the comparative analysis of the two prevailing electrode shapes employed in coplanar capacitive techniques—namely, triangular [34,41,43,48,52,53,66,67,72,76,78,79] and rectangular [35,38,40,44,49,68,80,81,82,83,84,85]—was conducted. Through a comprehensive synthesis of FE modeling simulations and empirical experimentation in [42], the analysis led to the conclusion that while both electrode shapes exhibit roughly equivalent penetration depths, the rectangular probe yields a slightly higher output signal. Additionally, it discovered that when two defects are close together, they can distort each other’s electric fields, potentially affecting measurement accuracy. However, this interference was significantly reduced when using rectangular electrodes. Therefore, the study recommended using paired rectangular electrodes, especially in situations where sensor size is limited. This configuration is particularly beneficial for multi-electrode capacitive sensor setups.

### 5.2. Dimension of the Electrodes

One of the principal design parameters influencing the performance of coplanar capacitive sensors is the size of the electrodes, which varies depending on the specific application. Small electrodes, while inadequate for scanning large defects, may prove insufficiently sensitive for detecting defects significantly smaller than their dimensions. Conversely, larger electrodes generally afford greater penetration depth and signal strength, albeit at the cost of reduced image resolution in imaging applications, owing to the sensor’s sampling of a larger volume [35]. Significantly, the optimization of signal-to-noise ratio stands as a paramount concern in capacitive sensor design [75]. Hence, a meticulous selection of electrode size is imperative to achieve optimal penetration depth and output signal across diverse applications. This aspect is extensively examined in [42]. Accordingly, a delicate balance must be struck between output signal, sensing depth (the extent of electric field penetration into the specimen), and electrode size, with optimization predicated upon the specific application context. Moreover, the overarching constraint of the sensor’s overall dimensions necessitates consideration [40,43,52].

### 5.3. Electrode Spacing

In the designing of a coplanar capacitive sensor, the spatial arrangement between the driving and sensing electrodes emerges as a pivotal determinant influencing penetration depth, electric field intensity, and overall sensor efficacy. This spatial configuration, termed the electrode spacing, delineates the distance between the peripheries of adjacent electrodes. Generally, separation between electrodes engenders an increase in penetration depth, as electric field lines necessitate traversing a greater distance within the specimen to reach the sensing electrode [53,72,86]. Nonetheless, this augmentation concurrently diminishes electric field strength, attributable to the attenuated coupling between the driving and sensing electrodes resultant from their increased spatial disparity. Conversely, a diminished separation between electrodes fosters a more concentrated distribution of electric field lines at the surface of the specimen, thereby augmenting sensitivity to superficial defects. Hence, a delicate equilibrium must be achieved between electric field intensity and penetration depth to engender optimal sensor performance [42,50,63,66].

### 5.4. Quantity and Configuration of the Electrodes

The determination of the appropriate number of electrodes for a specific application is contingent upon the geometric attributes of the sample under examination and its pertinent physical parameters. In instances wherein capacitance measurements directly correlate with specimen distance, as encountered in proximity or displacement measurement applications, a solitary sensing element—such as an annular sensor—often suffices to deduce specimen proximity from capacitance readings. Conversely, for endeavors characterized by greater complexity, such as imaging or NDT applications, an array of electrodes proves more appropriate. Moreover, due consideration must be accorded to the spatial arrangement of the electrodes and the available system real estate for electrode placement [40,42,66,73]. A comprehensive exploration of this aspect is detailed in [50,87].

### 5.5. Shielding Plate

The challenge posed by parasitic fields originating from the surrounding environment constitutes a primary obstacle in the utilization of capacitive sensors. Proximity between components of electrical conductors or circuits engenders parasitic capacitance, or stray capacitance, attributable to disparate voltages among these entities. This voltage differential engenders an electric field distribution, thereby precipitating the accumulation of electric charge. Despite being inherent, such parasitic capacitance is generally undesirable and necessitates minimization to the fullest extent feasible. A shielding plate serves as an effective measure to mitigate undesirable electric fields, eradicate stray capacitance and noise emanating from extraneous sources within the sensing region, and redirect electric field lines primarily towards the specimen [40,43,77,88]. Positioned on the rear surface of the primary electrodes and typically grounded, this shielding plate assumes a crucial role in sensor performance and optimization. According to [42], employing a conducting shielding plate above the substrate of coplanar electrodes enhances penetration depth while concomitantly diminishing the output signal. This attenuation in signal strength stems from the shielding plate’s absorption of electric field lines emanating from the primary electrodes, thereby attenuating the resultant output signal. Nonetheless, the advantageous attributes of employing a shielding plate supersede the reduction in the output signal, as it effectively mitigates the deleterious effects of parasitic capacitance and extraneous ambient fields.

### 5.6. Guard Electrode

The fundamental function of the guard electrode resides in diverting electric field lines away from direct access to the sensing electrode, thereby necessitating their traversal through the specimen to attain the sensing electrode, consequently augmenting penetration depth. Moreover, the guard electrode serves to mitigate noise originating from external sources and preclude direct coupling between driving and sensing electrodes. Conventionally, the guard electrode is maintained at ground potential [43], a configuration that engenders heightened penetration depth by confining the electric field within a more delimited spatial domain, thereby compelling field lines towards the tested material and amplifying penetration depth. Nevertheless, the presence of a guard electrode entails a concomitant reduction in the output signal, as it absorbs electric field lines emanating from the primary electrodes, thereby attenuating signal strength. The utilization of a guard electrode is particularly advantageous for the detection of deeper defects, as it effectively extends the penetration depth of the electric field into the specimen, enhancing the sensor’s capability to discern defects situated at greater depths within the material [35,42,63].

### 5.7. Lift-Off

Within the domain of coplanar capacitive sensing, the separation between the specimen surface and probe surface, commonly referred to as lift-off, stands as a pivotal consideration, bearing significant implications for sensor performance. Notably, an expansive lift-off presents a formidable impediment to this technique, as it engenders a reduction in capacitance between electrodes, thereby precipitating a commensurate diminution in output signal [79]. Moreover, an augmented lift-off curtails the extent to which the electric field penetrates the specimen, consequently diminishing penetration depth. This phenomenon underscores a critical challenge: a sizable lift-off can impede the detection of certain defects, particularly those situated at greater depths, as variations in electric field strength with depth manifesting closer to the surface. Consequently, an optimal sensor performance necessitates maintaining coplanar capacitive sensors at a minimal distance from the surface of non-conducting specimens, thereby maximizing output signal and penetration depth [42,79,89].

### 5.8. Frequency

In [42], the relationship between output signal and penetration depth was investigated across various excitation frequencies ranging from 0.1 kHz to 200 kHz, with all other parameters held constant. This examination aimed to elucidate the impact of excitation frequency on penetration depth and output signal when applied to non-conducting materials. Findings revealed distinctions between capacitive techniques and other electromagnetic NDT methodologies, such as eddy currents, wherein penetration depth is contingent upon the frequency of the input signal. Although frequency does not prominently influence capacitive sensor performance and exhibits negligible impact on penetration depth within the range of 10 to 200 kHz, it does exert discernible effects on the output signal within a limited scope. Notably, the frequency’s influence is contingent upon the electrical conductivity properties of the material under inspection and the specific instrumentation employed during experimentation.

The relative importance of performance indicators for coplanar capacitive sensing technology is summarized in Table 1.

## 6. Utilizing Coplanar Capacitive Sensing for Defect Detection Applications

This section provides a succinct overview of the diverse applications wherein the coplanar capacitive sensing technique is employed for the detection of varied defect types across an array of structures and components, facilitating the assessment of their structural integrity. Through a comprehensive review of the extant literature on this technique, it becomes apparent that its utilization in NDT is relatively nascent and evolving. Consequently, ongoing research endeavors primarily seek to elucidate and demonstrate the efficacy and potential of this technique across diverse applications. The utility of the coplanar capacitive sensing technique manifests in two primary categories: non-imaging and imaging applications, as delineated in [40]. Non-imaging applications entail defect identification through an observation of signal alterations detected by the probes. In contrast, imaging applications employ advanced data extraction and processing techniques to transform sensor data into visual representations, thereby revealing the presence of defects. This imaging capability enables detailed defect characterization, including an assessment of their spatial location, morphology, and dimensions.

*Reinforced Concrete (RC) Structure*: The RC structure is a critical construction material, and various NDT techniques have been explored to assess the condition of concrete structures and provide insight into their structural integrity. The authors have conducted detailed research on inspecting concrete samples using the capacitive imaging technique [75,90]. Experimental outcomes employing capacitive imaging probes underscored the viability of this technique in identifying surface cracks, sub-surface air voids, and steel reinforcement bars within concrete samples. Furthermore, line scans discussed in [82,91,92,93,94] demonstrated the efficacy of coplanar capacitive sensors in detecting varying sizes/depths of reinforcement bars embedded within concrete specimens. These results affirmed the sensor’s efficacy in detecting defects, metal bars, and their structural features across concrete.

Dérobert et al. [38] explored moisture assessment in cover concrete, contrasting the coplanar capacitive technique with ground penetrating radar (GPR). Their efficacy was scrutinized through laboratory experiments on various test slabs. The study highlighted the capacitive probe’s capability to inspect cover concrete across customizable depths, unlike GPR. Moreover, the probe’s direct output simplifies interpretation and correlation with water content, bypassing the need for offline calculations or coring.

In [90,95], the capacitive imaging technique was employed to examine a concrete sample containing a concealed channel with varying depths. A stepped air-filled channel was incorporated through the center to simulate a local change in thickness, resembling the presence of a void when viewed from the flat top surface. The resulting image showed lighter areas indicating the presence of both shallow and deep voids. Additionally, the result revealed that the shallower channel produced a lower output than the region containing the deeper channel. This suggests that depth information can be gleaned from such scans, showcasing the potential of capacitive imaging in assessing concrete structures and identifying anomalies like voids.

*Glass Fiber Reinforced Polymer (GFRP)*: In [41], a GFRP plate, which featured three pre-existing slots with rectangular cross-sections, was investigated using a coplanar capacitive probe. These slots were strategically located to simulate delaminations and water intrusion defects at various depths within the composite plate. Initially, the plate underwent testing with the slots left empty to mimic delamination defects. Subsequently, the plate was tested after the slots were injected with water, completely filling and sealing them to replicate water intrusion defects. According to the test results, the measured capacitances reflected the presence of delaminations, with capacitance increasing proportionally to the depth of the delamination. Conversely, in the case of water intrusion defects, capacitance measurements exhibited a decrease relative to the depth of the defect.

In [41,76], the investigation focused on two pultruded glass-fiber-reinforced polymer composite specimens that experienced impact damage [41,76] and contained a crack [41]. For the examination of these samples, a triangular probe was employed for imaging purposes. The examination revealed variations in image coloration, effectively delineating regions of damage. Subsequently, the utilization of a coplanar capacitive probe facilitated the identification of damaged areas within the composite specimens, demonstrating the imaging technique’s sensitivity to alterations in dielectric properties induced by material damage.

*Sandwich Structure*: According to [41], the application of capacitive imaging extended to the examination of sandwich structures. It is imperative to note that, owing to the capacitive nature of the technique, the probing electric field may not adequately penetrate conducting faces, such as those crafted from aluminium and carbon fiber. Consequently, the method finds optimal utility in sandwich structures featuring non-conducting face sheets, typically composed of GFRP. A series of experiments were conducted to validate the applicability of the CI technique for inspecting sandwich structures featuring an aluminum honeycomb core, with a glass fiber composite panel positioned atop. The primary objective was to identify structural folding failures within the aluminum honeycomb structure and discern cells filled with oil and water. The results demonstrated effective detection of the folding failure, evident as a lighter area in the image. Additionally, cells filled with fluid were successfully identified, with oil intrusion appearing darker and water intrusion appearing lighter in the image. These findings highlight the utility of capacitive imaging as an additional technique for detecting fluid intrusion, providing non-contact and contamination-free detection from a single surface.

*Carbon Fiber Reinforced Polymer (CFRP)*: As previously discussed in the previous sections, this electric field from the CI probe establishes an equipotential surface on carbon fiber composites, owing to their relatively high electrical conductivity. Consequently, this tends to obscure sub-surface features from detection. Thus, in such cases, the CI technique demonstrates heightened sensitivity to surface features. The response of capacitive imaging probes to impact damage on laminated carbon fiber composite samples was examined, and the results depicted the extent of impact damage. To further investigate internal structural changes, a scan using an air-coupled ultrasound transducer pair in through-transmission mode was conducted on the same sample. Remarkably, the areas identified in the ultrasonic scans exhibited a strong correlation with the impact damage areas identified from the CI scans [41]. In addition, in [49], capacitive images were obtained of drilled holes within a carbon fiber reinforced polymer (CFRP) slab and of delamination resulting from impact in various CFRP samples. These images provide valuable insights into the structural integrity and damage characteristics of the material, assisting in the assessment and improvement of CFRP-based components in engineering applications.

*Perspex and Plexi-glass*: Capacitive imaging has been employed to investigate Perspex [35,43,67,76] and Plexi-glass [52], revealing both surface and hidden defects. Perspex serves as an insulating dielectric material. The resulting image illustrates the sensitivity of the capacitive imaging technique to both surface and subsurface defects in the specimens, albeit with varying resolutions. This non-destructive technique allows for a comprehensive examination of the dielectric materials, providing insights into the nature and extent of defects present. Such findings contribute to enhancing the understanding of material quality and can inform improvements in manufacturing processes for Perspex and Plexi-glass components across various applications.

*Conductive Material*: Capacitive imaging scans were meticulously performed on conductive specimens, including aluminum [34,35,43,79] and steel [43] plates, showcasing an array of surface defects spanning varying depths. The outcomes of these scans vividly depict the imaging of these defects, where their depths align precisely with the intensity of the captured images. This correlation between defect depth and image intensity not only validates the efficacy of capacitive imaging in flaw detection but also underscores its potential for precise quantification of defects within metallic surfaces. Such detailed insights garnered from this study are instrumental in advancing non-destructive testing methodologies, thereby enhancing the reliability and integrity of metallic structures across diverse industrial sectors.

*Hybrid Structure*: Scans were conducted on an aluminum plate featuring surface defects of varying depths, with the surface coated with a layer of insulation as described in [35,68,84,91]. Both imaging [35,68] and line scans [84,91] revealed the detection of holes, with the detected amplitude corresponding to hole depth. In [48,95], experiments were conducted to evaluate the effectiveness of the coplanar capacitive technique in delineating surface features on a steel plate through insulation. A segment of high-density extruded polystyrene, commonly known as Styrofoam, was precisely machined into a plate with varying thicknesses. This polystyrene plate was positioned atop the same steel plate utilized in prior investigations to simulate diverse insulation layer depths. The analysis revealed that, with the increasing thickness of the insulation layer, the output signal demonstrated a decrease. This outcome exhibits promise for surface feature detection in metals beneath insulation coatings, which can be developed for characterizing corrosion under insulation (CUI).

*Other Materials and Structures*: [75] unveiled the capability of the capacitive imaging technology to penetrate common building materials such as plywood sheet. The assessment of the sensor’s effectiveness in subsurface imaging involved placing two metal bars vertically and horizontally behind a plywood sheet. An analysis of the generated 2D subsurface image revealed a precise depiction of the metal bars’ positions and orientations. These findings confirm the sensor’s ability to accurately locate and characterize the metal bars, providing valuable insights into the concealed subsurface behind the plywood sheet. Wooden studs play a significant role in the construction of buildings, often concealed within walls, making their detection challenging. To address this, the capacitive sensor was tested on a wall to assess its ability to locate wooden studs and determine their orientations. The subsurface image distinctly revealed the location and orientation of the stud. These findings underscore the sensor’s effectiveness in detecting studs and discerning their orientations with precision.

Genest et al. [72] discussed the utilization of planar capacitive probes to assess the misalignment of ceramic tiles, specifically in two-dimensional array arrangements commonly found in armored vehicles. Ceramic tiles serve as protective layers against projectile penetration in military and security contexts. Ensuring minimal misalignment, such as inter-tile gaps and out-of-plane mismatch, is crucial for effective protection. The study involves the investigation of linear arrays of rectangular alumina tiles using three planar capacitive probes. The research successfully discriminated inter-tile gaps as small as 0.5 mm and out-of-plane tile misalignment down to 0.25 mm, highlighting the effectiveness of the capacitive probes in assessing tile alignment in armored vehicle applications.

Wang et al. [71] addressed the difficulty of inspecting composite sucker rods, which pose challenges for conventional non-destructive evaluation (NDE) methods because of their intricate material composition and structure. The study explored the feasibility of using capacitive imaging (CI) for this purpose. Preliminary CI experimental results demonstrated the detection of surface wear defects in the glass-fiber layer and the acquisition of the carbon core profile. Additionally, a series of accelerated aging experiments suggested that CI could potentially assess the aging status of composite sucker rods. According to this research, the CI technique holds promise for addressing challenging tasks in the non-destructive evaluation of composite sucker rods, offering a valuable tool for ensuring their integrity and longevity in various applications.

In [85], the discontinuity of electrical properties at adhesive bond interfaces was investigated by capacitive imaging. The presence of defective adhesive bonds poses significant risks to structural integrity due to compromised joint strength. Despite its critical importance, the fundamental nature of the adhesion process between two solids remains inadequately understood, primarily because adhesion phenomena permeate numerous scientific and technological domains. Of particular interest from the perspective of NDT is the concept of discontinuous properties within adhesion interfaces. These discontinuities are heavily contingent upon the quality of the interface formed between the adhesive and substrate. Capacitive imaging (CI) emerges as a promising avenue in this regard. This technique holds particular promise as adhesives and substrates typically exhibit disparate dielectric properties. At the adhesive–substrate interface, discontinuities in dielectric properties induce abrupt alterations in the spatial distribution of electric fields, consequently influencing capacitance measurements. Such alterations effectively simulate defects within adhesive joints, exacerbating uncertainties in permittivity assessments. This study not only elucidated how capacitance measurements reflect electric field distribution but also furnished insights into bond strength.

In [96], the capacitive imaging (CI) technique was employed to image non-metallic objects concealed within cylindrical media. The experimental results presented in this paper serve to underscore the performance and efficacy of the CI technique in accurately detecting and imaging objects within cylindrical non-metallic media. Furthermore, recent advancements in CI technology have highlighted its promising utility as a reliable method for inspecting cylindrical non-metallic media. Building upon these developments, this study leveraged CI to enhance the imaging of concealed objects, particularly within cylindrical environments. The application of CI in this context represents a significant advancement in non-destructive testing methodologies, offering a versatile and efficient solution for inspecting cylindrical non-metallic media. Through comprehensive experimentation and analysis, the efficacy of CI has been demonstrated as a valuable tool for industries and research fields requiring precise imaging capabilities in cylindrical environments. This research contributes to the growing body of knowledge surrounding CI applications, further solidifying its position as a promising technique for a wide range of imaging tasks, particularly those involving cylindrical non-metallic media.

The aforementioned applications demonstrate the vast potential of the coplanar capacitive sensing technique for numerous other applications that warrant exploration in the future. These results suggest specific advantages and potential benefits of capacitive imaging for industrial applications. Ongoing research aims to explore its utility in challenging scenarios like corrosion under insulation (CUI), impact damage in foam-cored composites, and other industrial concerns typically difficult to assess with conventional methods due to scattering and attenuation. From the discussed applications, it becomes apparent that all sensor designs can be employed for both non-imaging and imaging applications. Typically, the selection between non-imaging and imaging approaches in NDT is dictated by the specific purpose of the inspection. Non-imaging methods focus solely on detecting the presence of defects. While some defect characteristics can be inferred from sensor data, they often lacks the depth required for comprehensive defect characterization. Thus, if the goal is to thoroughly characterize defects in terms of size, shape, and location, an imaging approach is necessary. In the development of various sensor designs for different applications, studies have predominantly focused on ascertaining the sensor’s capability for a particular task, followed by optimization endeavors to enhance performance, often achieved through adjustments to design parameters.

## 7. Benefits and Limitations of Coplanar Capacitive Sensors

Traditional contact sensors, often used in applications such as pipeline inspection, aircraft maintenance, and bridge structural monitoring, require direct contact with the surface, leading to operational challenges such as insulation removal, precise sensor placement, and labor-intensive installations. For instance, in pipeline inspection, contact sensors necessitate insulation removal and surface preparation, causing significant operational downtime. Similarly, in aircraft maintenance, achieving precise placement on complex, curved surfaces requires substantial manual effort, while in bridge monitoring, installing sensors on large and hard-to-reach areas can be both costly and time-consuming [97]. In contrast, coplanar capacitive sensors offer significant advantages by enabling non-contact, rapid inspections even through insulating materials, adapting to complex geometries, and providing comprehensive coverage with minimal manual intervention. These sensors can inspect pipelines without the need for direct contact, thereby minimizing downtime and operational disruptions. In the aerospace industry, coplanar capacitive sensors facilitate quick, non-contact inspections that enhance the efficiency and accuracy of maintenance routines. For bridge structural monitoring, these sensors allow for continuous, real-time monitoring without direct contact, simplifying installation and maintenance processes [1].

The capacitive technique presents a promising avenue for mitigating certain constraints inherent in conventional NDT methodologies. Notably, this technique operates through volume averaging, eliminating the scattering issues associated with ultrasound methods [41]. Characterized by cost-effectiveness and adaptability to specific applications, capacitive sensors offer a spectrum of advantages. They boast rapid response times, non-intrusive and non-invasive capabilities, radiation-free operation, and flexible electrode design possibilities. Moreover, the coplanar structure facilitates single-sided specimen interrogation, particularly beneficial in scenarios where access to the specimen is restricted [40,42,46,50]. Furthermore, being a non-contact technique, capacitive sensing eliminates the need for direct physical contact with the specimen [44,92], allowing for the presence of lift-off, which holds potential for applications such as corrosion detection under insulation (CUI), including the identification of surface imperfections in metals and even minute rust formations [36,48,91]. Additionally, the fringing electric fields emitted by coplanar electrodes penetrate non-conductive materials, facilitating measurements on surfaces with external coatings or paint layers. These versatile attributes render coplanar capacitive sensors highly sought-after in a plethora of applications encompassing proximity/displacement measurement [51], NDT [34], material characterization [63], and imaging [52,98]. Nonetheless, coplanar capacitive sensors are generally accompanied by relatively few limitations. One notable drawback of this technique is its susceptibility to sizable lift-off distances. Increased air gaps between the specimen surface and the probe result in diminished measurement accuracy and reduced sensitivity of the sensor to deeper flaws [42]. Furthermore, capacitive sensors rely on induced electric fields, thereby restricting measurements to non-conductive or partially conductive materials. Consequently, the electric field emitted by coplanar capacitive sensors cannot permeate conductive materials, thereby limiting their utility in inspecting the internal features of such materials [36]. Table 2 summarizes the advantages and limitations of the coplanar capacitive sensing method.

## 8. Conclusions

This paper provides a comprehensive examination of the coplanar capacitive sensing technique, covering fundamental principles, performance factors, diverse designs, and current applications, primarily focused on defect detection. Its foundational concepts underpinning the operation are well-documented and straightforward, facilitating accessibility for newcomers to the field. The technique performance hinges on sensitivity, penetration depth, and signal strength, which are significantly influenced by various factors, particularly the geometric parameters of the sensor. Consequently, optimizing sensor designs for different applications primarily involves adjusting geometric parameters. While the coplanar capacitive sensing technique offers numerous advantages, such as the ability to detect defects in both conducting and non-conducting materials, as well as evaluating composites with complex matrix structures, challenges persist within coplanar capacitive sensing techniques. These challenges include unavoidable trade-offs during sensor optimization, the complexity of analytical models, lift-off effects, and the need for application-specific optimization techniques. To address these challenges and advance the technique, further research is essential. Future research directions identified in the review include determining the exact size of defects, improving performance through enhanced sensor circuitry and materials, capacitive mode enhancement for dual-mode sensors, exploring different fabrication techniques and materials, and discovering new applications. Addressing the challenges mentioned and exploring these future research areas will set a strong foundation for developing the coplanar capacitive sensing technique for the next generation of non-destructive testing and evaluation. Our future work will focus on tackling the issues outlined in this review and investigating the suggested research directions. This aims to improve and advance the coplanar capacitive sensing technique in non-destructive evaluation.

## Figures and Tables

**Table 1:** Relative importance of performance indicators for coplanar capacitive sensing technique [42].

Factor	Key Parameters	Impact on Performance
Geometry of	Various shapes	Rectangular: Higher output signals,
Electrodes		reduced defect interference.
		Other shapes: Different electric field
		distributions, sensitivities.
Dimension of	Electrode size	Larger: Greater penetration depth and
Electrodes		signal strength; lower image resolution.
		Smaller: Lower penetration depth;
		higher resolution.
Electrode Spacing	Distance between	Applicable to any metal
	electrodes	Greater: More penetration depth; lower
		signal strength.
		Closer: Enhanced surface defect sensitivity.
		sensitivity.
Quantity and	Number and	More: Suitable for complex
Configuration	arrangement of	tasks (e.g., imaging).
	electrodes	Arrangement: Consider spatial
		placement for optimal performance.
Shielding Plate	Position and grounding	Reduces parasitic capacitance,
		stray noise.
		Increases penetration depth and
		reduces output signal. Grounded, rear
		position.
Guard Electrode	Grounded guard	Redirects field lines,
	electrodes	enhances penetration depth.
		Reduces noise. Absorbs field lines,
		and reduces output signal.
Lift-off	Distance from	Greater: Reduces capacitance,
	probe to specimen	output signal, and penetration depth.
		Minimal for optimal performance.
Frequency	Excitation frequency	Minimal effect on penetration
	(0.1 kHz to 200 kHz)	depth, slight influence
		on the output signal.
		Depends on material conductivity
		and instrumentation.

**Table 2:** Summary of advantages and limitations of the coplanar capacitive sensing method.

Advantages	Limitations
- Volume averaging [41]: Eliminates scattering issues of ultrasound methods - Cost-effectiveness and adaptability [40,42,46,50]: Suitable for specific applications Rapid response times Non-intrusive and non-invasive Radiation-free operation Flexible electrode design Single-sided specimen interrogation - Non-contact technique [44,92]: Allows for lift-off presence detection - Penetration of non-conductive coatings [36,48,91]. - Versatile in proximity/displacement measurement, material characterization, and imaging [34,51,52,63,98].	- Susceptibility to lift-off distances [42]: Decreased accuracy with air gaps - Limited to non-conductive materials [36]: Cannot penetrate conductive materials

## Data Availability

Not applicable.
